# Supplementary material for: The Helicobacter pylori Genome Project: insights into H. pylori population structure from analysis of a worldwide collection of complete genomes
Source: Nat Commun. 2023 Dec 11;14:8184. doi: 10.1038/s41467-023-43562-y (PMC10713588; doi:10.1038/s41467-023-43562-y)

Supplementary information:

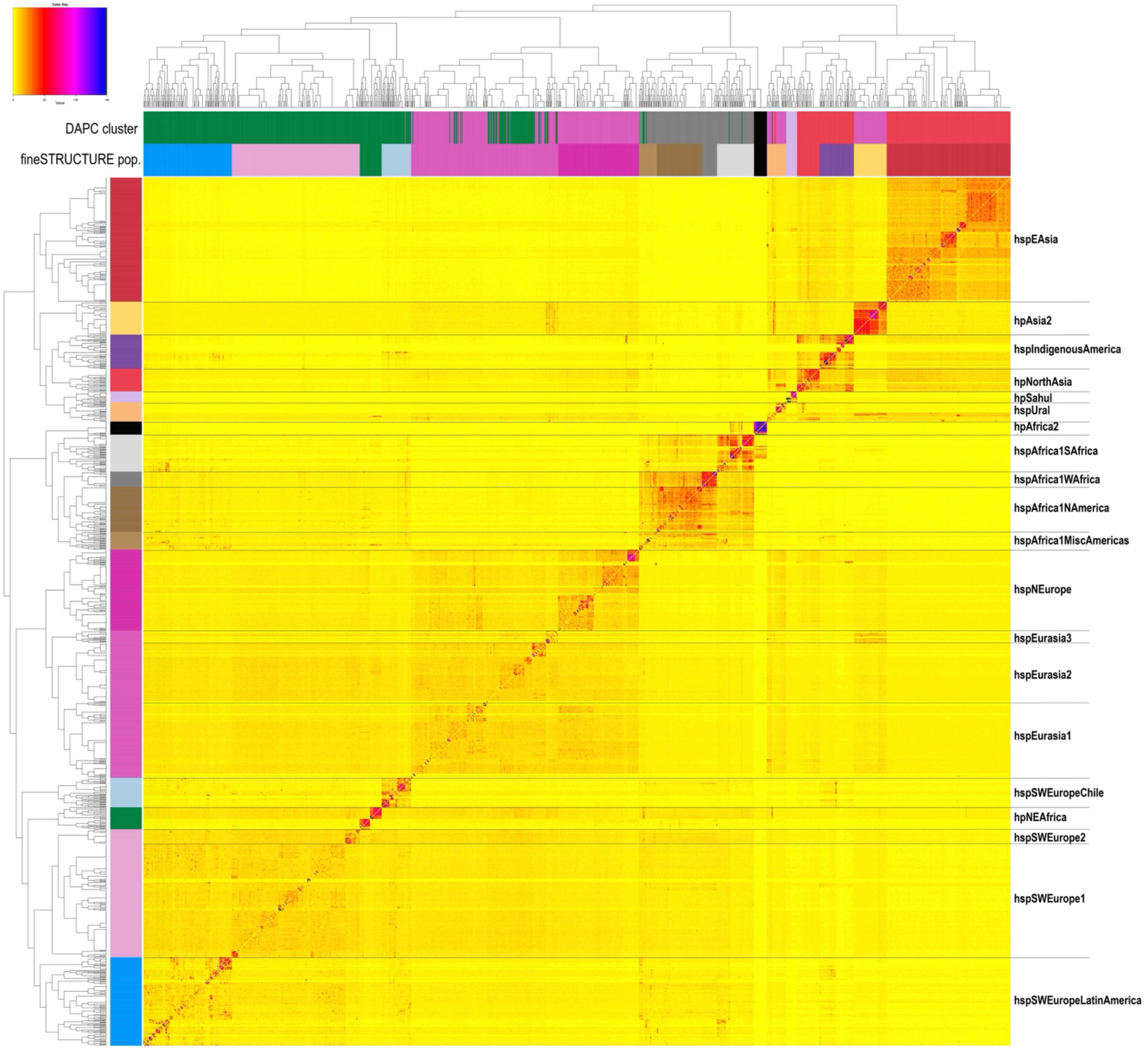

Supplementary Figure 1.

Population structure of global *H. pylori* strains.

The colour of each cell of the matrix indicates the expected number of DNA chunks imported from a donor genome (column) to a recipient genome (row). The inferred tree was generated by Bayesian clustering in fineSTRUCTURE. The colour bars on the top and left indicate suggested *H. pylori* population (hp) and subpopulation (hsp) as in Fig. 1, and the discriminant analysis of principal components, DAPC, K=6 cluster (Fig S3), respectively.

Supplementary Figure 2. fineSTRUCTURE dendrogram with indicated population labels and colours. The plot is including chromosome painting proportions (vertical bars) for each genome. The colours indicate *H. pylori* population (hp) and subpopulation (hsp) assignments.

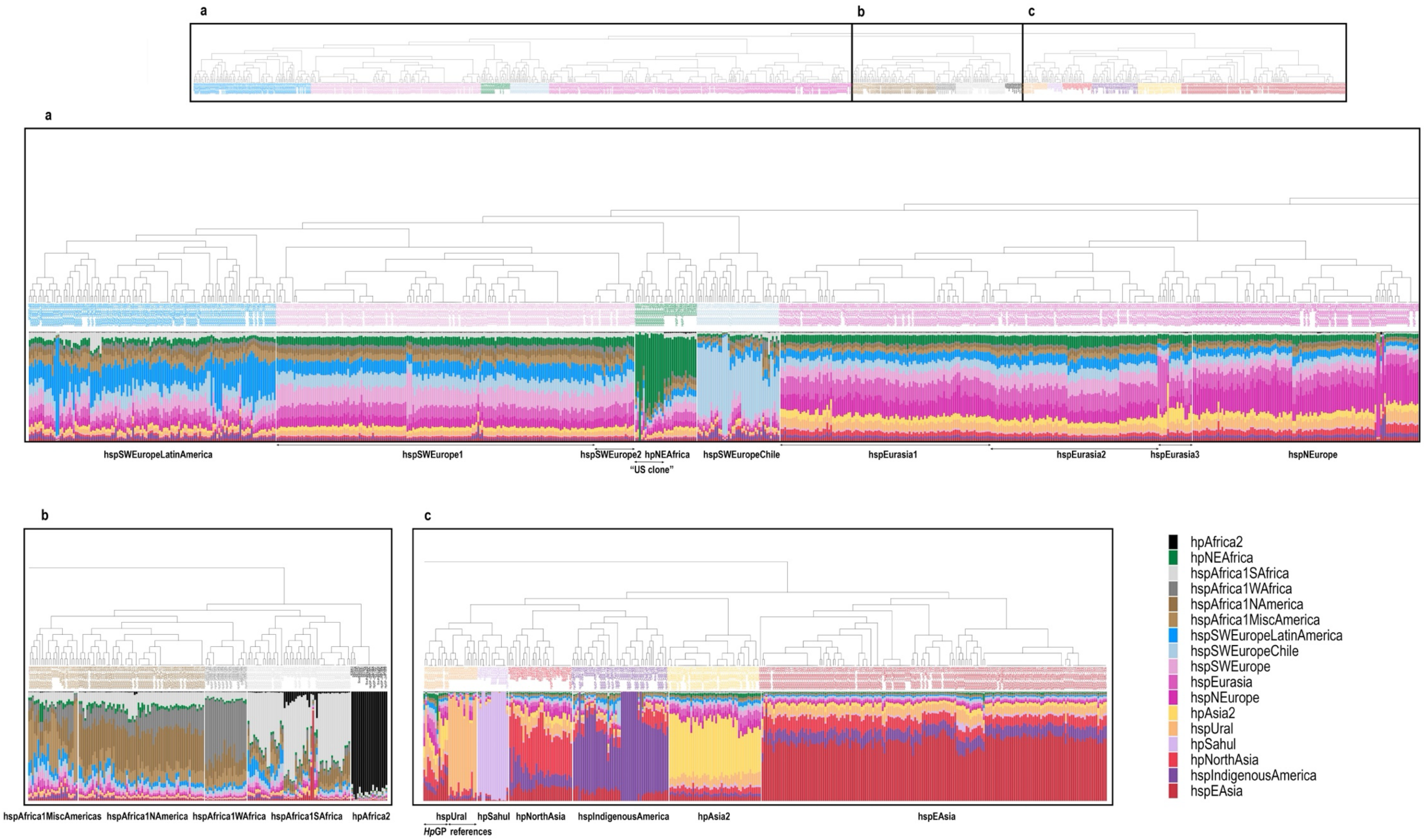

Supplementary Figure 3. Discriminant analysis of principal components (DAPC) employing K = 6 genetic clusters.

a) The map reports geographic distribution and frequency of individuals belonging to each of the 6 clusters. b) DAPC scatterplot for the analysis including all samples, c) DAPC scatterplot excluding the genomes of the outlier groups (group 4 and 6). d) Barplot representing the posterior group membership probability of each sample to actually belong the group assigned by the *find.cluster* procedure.

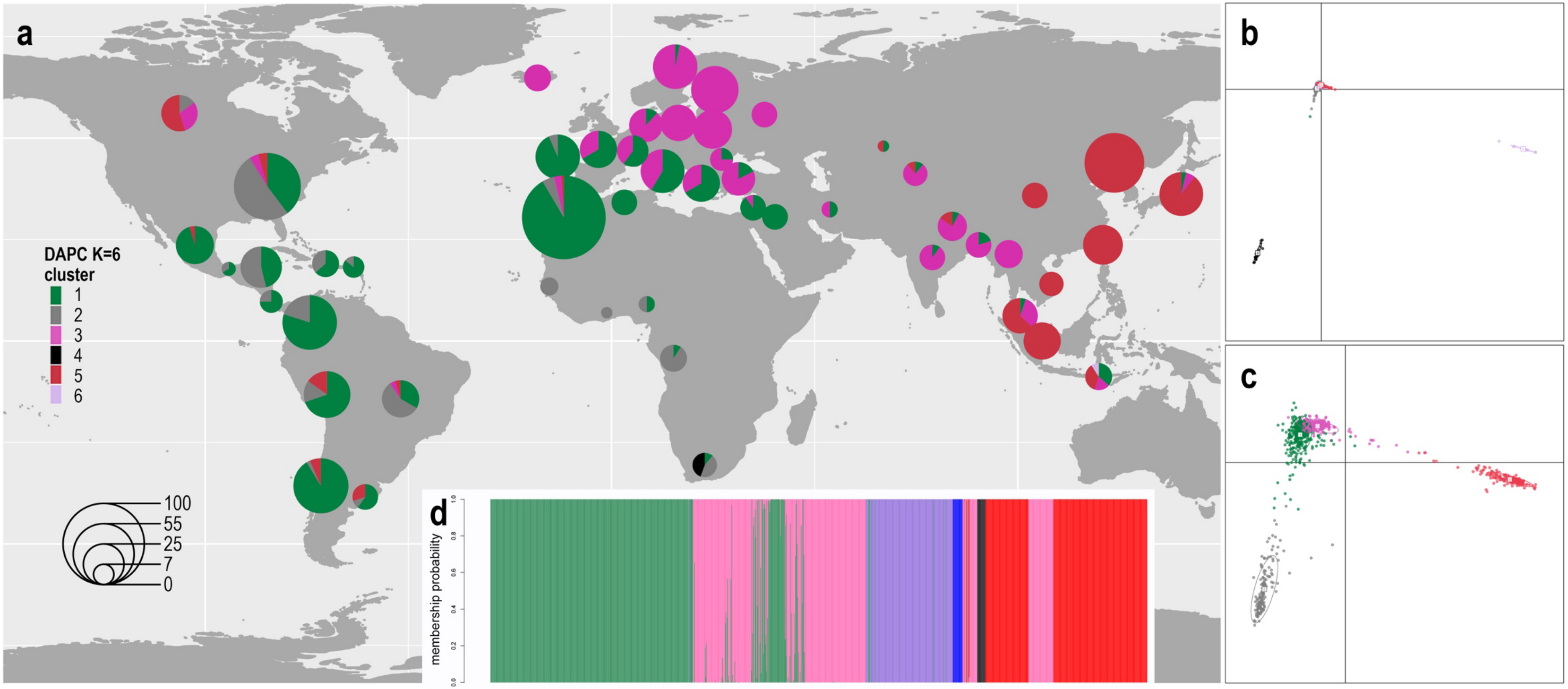

Supplementary Figure 4. Discriminant analysis of principal components (DAPC) employing K = 17 genetic clusters.

a) The map reports geographic distribution and frequency of individuals belonging to each of the 17 clusters. b) DAPC scatterplot for the analysis including all samples, c) DAPC scatterplot excluding the genomes of the outlier groups (group 4 and 17). d) Barplot representing the posterior group membership probability of each sample to actually belong the group assigned by the *find.cluster* procedure.

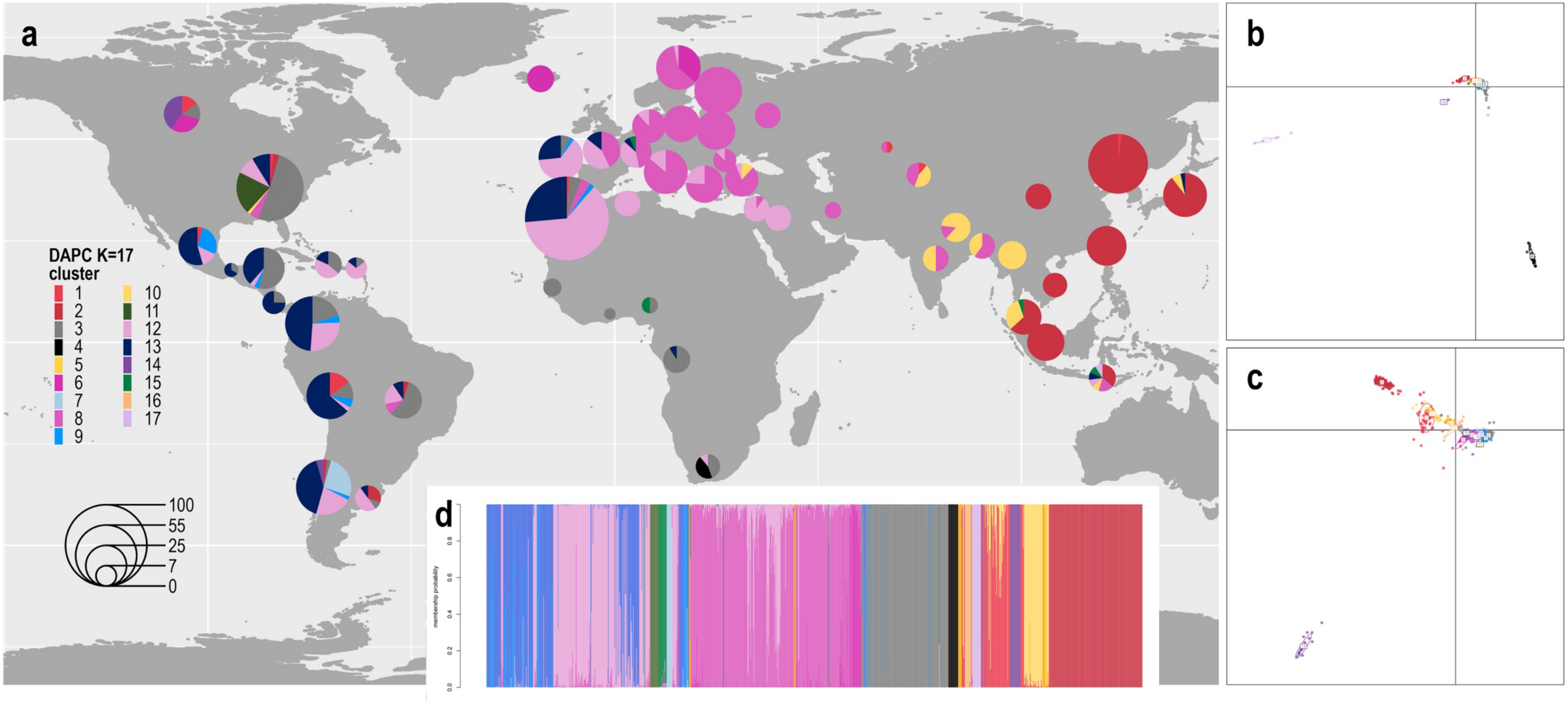

Supplement: Supplementary file 1 — Supplementary Information [file 41467_2023_43562_MOESM1_ESM.pdf]
