# Supplementary material for: The Helicobacter pylori Genome Project: insights into H. pylori population structure from analysis of a worldwide collection of complete genomes
Source: Nat Commun. 2023 Dec 11;14:8184. doi: 10.1038/s41467-023-43562-y (PMC10713588; doi:10.1038/s41467-023-43562-y)
Supplement: Supplementary file 3 — Description of Additional Supplementary Files [file 41467_2023_43562_MOESM3_ESM.pdf]

Description of Additional Supplementary Files:

Supplementary Data 1: Details of the 1,012 genomes sequenced within HpGP, and their genome statistics

Supplementary Data 2: Details of the 255 representative genomes included for population structure reference.

Supplementary Data 3: Detailed geographical origin and subpopulation assignments of the entire HpGP set.

Supplementary Data 4: Table of public US genomes included in the in-depth analysis of the highly clonal US cluster.
