# Supplementary material for: The Helicobacter pylori Genome Project: insights into H. pylori population structure from analysis of a worldwide collection of complete genomes
Source: Nat Commun. 2023 Dec 11;14:8184. doi: 10.1038/s41467-023-43562-y (PMC10713588; doi:10.1038/s41467-023-43562-y)
Supplement: Supplementary file 8 — Reporting Summary [file 41467_2023_43562_MOESM8_ESM.pdf]

nature portfolio

Corresponding author(s): Kaisa Thorell

Last updated by author(s): Oct 29, 2023

Reporting Summary

Nature Portfolio wishes to improve the reproducibility of the work that we publish. This form provides structure for consistency and transparency in reporting. For further information on Nature Portfolio policies, see our Editorial Policies and the Editorial Policy Checklist.

Statistics

For all statistical analyses, confirm that the following items are present in the figure legend, table legend, main text, or Methods section.

n/a	Confirmed	
☐	☒	The exact sample size (*n*) for each experimental group/condition, given as a discrete number and unit of measurement
☐	☒	A statement on whether measurements were taken from distinct samples or whether the same sample was measured repeatedly
☐	☒	The statistical test(s) used AND whether they are one- or two-sided<br>*Only common tests should be described solely by name; describe more complex techniques in the Methods section.*
☐	☒	A description of all covariates tested
☐	☒	A description of any assumptions or corrections, such as tests of normality and adjustment for multiple comparisons
☐	☒	A full description of the statistical parameters including central tendency (e.g. means) or other basic estimates (e.g. regression coefficient) AND variation (e.g. standard deviation) or associated estimates of uncertainty (e.g. confidence intervals)
☒	☐	For null hypothesis testing, the test statistic (e.g. *F*, *t*, *r*) with confidence intervals, effect sizes, degrees of freedom and *P* value noted<br>*Give P values as exact values whenever suitable.*
☐	☒	For Bayesian analysis, information on the choice of priors and Markov chain Monte Carlo settings
☐	☒	For hierarchical and complex designs, identification of the appropriate level for tests and full reporting of outcomes
☒	☐	Estimates of effect sizes (e.g. Cohen's *d*, Pearson's *r*), indicating how they were calculated

Our web collection on statistics for biologists contains articles on many of the points above.

Software and code

Policy information about availability of computer code

Data collection	No software was used to collect the data.
Data analysis	Genome Assembly<br>SMRTLink's HGAP4/Microbial Assembly<br>Hifiasm v0.13-r308<br>circlator v1.5.3<br>MUMmer v3.23<br>prokka v1.14.6<br>BUSCO v5.4.7<br>NCBI Prokaryotic Genome Annotation Pipeline, PGAP https://www.ncbi.nlm.nih.gov/genome/annotation_prok/)<br>panaroo pipeline v1.2.10<br>BEAGLE v.3.3.2<br>fineSTRUCTURE v4<br>ChromoPainter v2<br>SNP-sites v.2.5.1<br>PAUP v.4.0a166<br>chewBBACA software v2.8.5<br>Prodigal v2.6.3<br>GrapeTree v1.5.0

mash v2.3
PhyML v3.1
ClonalFrameML v1.11-3-g4f13f23

R packages:
adegenet
compoplot
BactDating
ggplot2
ggmaps
plotly

The computational scripts to process the data and plot figures are available at https://github.com/HpGP/Code-and-Data v1.0. This code is also archived on Zenodo under DOI 10.5281/zenodo.8381170.

For manuscripts utilizing custom algorithms or software that are central to the research but not yet described in published literature, software must be made available to editors and reviewers. We strongly encourage code deposition in a community repository (e.g. GitHub). See the Nature Portfolio guidelines for submitting code & software for further information.

Data

Policy information about availability of data

All manuscripts must include a data availability statement. This statement should provide the following information, where applicable:

- Accession codes, unique identifiers, or web links for publicly available datasets
- A description of any restrictions on data availability
- For clinical datasets or third party data, please ensure that the statement adheres to our policy

The whole genome sequences generated within the HpGP have been deposited in the NCBI GenBank database under BioProject accession code PRJNA529500 [https://www.ncbi.nlm.nih.gov/bioproject/ PRJNA529500] (Supplementary data 1). NCBI or equivalent public accessions for the reference set are listed in Supplementary Data 2. The whole HpGP genome dataset and the 255 reference genomes are also deposited to Zenodo, DOI 10.5281/zenodo.10048320. Source data for the individual figures are available in Supplementary Data 5.

Research involving human participants, their data, or biological material

Policy information about studies with human participants or human data. See also policy information about sex, gender (identity/presentation), and sexual orientation and race, ethnicity and racism.

Reporting on sex and gender	We only discuss bacterial genomes in the study and not age, sex, or gender of the individuals they were isolated from.
Reporting on race, ethnicity, or other socially relevant groupings	Helicobacter pylori shares co-evolutionary history with humans. This has led to the development of genetically distinct H. pylori subpopulations associated with the geographic origin of the host and to some extent to ethnicity within geographical areas. This way, even if we are only studying bacterial genomics, this is to a rather high extent an indicator of human origin. The most pronounced example is for Indigenous American peoples which due to their migration history followed by long periods of genetic isolation are carriers of H. pylori belonging to a distinct H. pylori population, termed hpIndigenousAmerica. Low socioeconomic status is tightly associated with both the prevalence of H. pylori infection and with elevated gastric cancer risk. However, in this manuscript we have only focused on bacterial genetics and not discussed the race, ethnicity or other groupings of the humans carrying the bacteria.
Population characteristics	Neither age, sex or disease diagnosis have been analysed in the paper.
Recruitment	The HpGP samples represent a convenient set. Contributors of samples were identified through advertisements at international scientific meetings, direct invitations to known colleagues and investigators with published sets of H. pylori strains, as well as referrals. A limited number of H. pylori genomes is publicly available from Spain, one of the main countries responsible for colonial activities in the Americas. Thus, in collaboration of members of the Spanish Association of Gastroenterology, we oversampled this country to better understand the admixed genomes from individuals from Latin American and the Caribbean.<br><br>In particular, we obtained gastric tissues (fresh frozen with and without culture media; n=351) and cultures (pooled or single colonies; n=660) of H. pylori from individuals with non-atrophic gastritis (n=606), advanced intestinal metaplasia (n=172 with extension to gastric corpus or incomplete-type restricted to antrum), and gastric cancer (n=233). Samples were collected between 1995 and 2020. All individuals provided informed consent, and local Institutional Review Boards approved sample collection. The HpGP was exempted from institutional review board evaluation by the National Institutes of Health Office of Human Subjects Research Protection. The summary statistics of 1011 included strains are presented in Table 1, and corresponding NCBI accession numbers and genome statistics are presented in Supplementary Information Table 1.
Ethics oversight	Ethical permissions have been granted by local ethical boards and all individuals contributing with bacterial strains have given informed consent.

Note that full information on the approval of the study protocol must also be provided in the manuscript.

Field-specific reporting

Please select the one below that is the best fit for your research. If you are not sure, read the appropriate sections before making your selection.

[x] Life sciences [ ] Behavioural & social sciences [ ] Ecological, evolutionary & environmental sciences

For a reference copy of the document with all sections, see nature.com/documents/nr-reporting-summary-flat.pdf

Life sciences study design

All studies must disclose on these points even when the disclosure is negative.

Sample size	1,011 bacterial genomes isolated from clinical specimens together with reference strain 26695 were sequenced and analysed, in total 1,012 genomes. In addition we included a reference set of 255 selected representative worldwide genomes. One of the primary aims of the HpGP is to conduct bacterial genome-wide association analyses of gastric cancer (n=233) and advanced intestinal metaplasia (n=172) cases vs. non-atrophic gastritis/mild atrophy controls (n=606). The two previous genome-wide association studies (GWAS) of gastric cancer strains have reported statistically significant associations with cases ranging from 49 (European-descent strains; Berthenet E et al., 2018) and 125 (Asian-descent strains; Tuan VP et al., 2021). In terms of gastric cancer cases (n=233), the HpGP set includes 83 European-descent strains and 132 Asian-descent strains. For the characterization of H. pylori populations, a small number of representative samples is sufficient. Our analysis of population structure within the HpGP significantly exceeds previous sample sizes, and allowed us to identified novel subpopulations.
Data exclusions	No genomic data were excluded from the analysis. The selection of the representative dataset is described in detail in the methods section.
Replication	1. A consolidated QC report is presented in Supplementary Data 1.<br>2. The HpGP samples represent a convenient set. Contributors of samples were identified through advertisements at international scientific meetings, direct invitations to known colleagues and investigators with published sets of H. pylori strains, as well as referrals. We achieved a high level of worldwide geographical coverage, however, some areas are still underrepresented, as we also discuss in the manuscript.
Randomization	Not applicable.
Blinding	We did not know beforehand which populations the different samples would group in.

Reporting for specific materials, systems and methods

We require information from authors about some types of materials, experimental systems and methods used in many studies. Here, indicate whether each material, system or method listed is relevant to your study. If you are not sure if a list item applies to your research, read the appropriate section before selecting a response.

Materials & experimental systems

n/a	Involved in the study	
[x]	[ ]	Antibodies
[x]	[ ]	Eukaryotic cell lines
[x]	[ ]	Palaeontology and archaeology
[ ]	[x]	Animals and other organisms
[x]	[ ]	Clinical data
[x]	[ ]	Dual use research of concern
[x]	[ ]	Plants

Methods

n/a	Involved in the study	
[x]	[ ]	ChIP-seq
[x]	[ ]	Flow cytometry
[x]	[ ]	MRI-based neuroimaging

Animals and other research organisms

Policy information about studies involving animals; ARRIVE guidelines recommended for reporting animal research, and Sex and Gender in Research

Laboratory animals	n/a
Wild animals	n/a
Reporting on sex	This has not been used as a parameter in our analysis.
Field-collected samples	n/a
Ethics oversight	The project only concerns work with bacterial strains for which ethical review is not required.

Note that full information on the approval of the study protocol must also be provided in the manuscript.

Plants

Seed stocks	n/a
Novel plant genotypes	n/a
Authentication	n/a
